# Features of urine S100B and its ability to rule out intracranial hemorrhage in patients with head trauma: a prospective trial

**DOI:** 10.1007/s00068-019-01201-6

**Published:** 2019-08-06

**Authors:** Tomas Vedin, Mathias Karlsson, Marcus Edelhamre, Mikael Bergenheim, Per-Anders Larsson

**Affiliations:** 1 Department of Clinical Sciences, Lund University, Svartbrödragränden 3-5, 251 87 Helsingborg, Sweden; 2 Department of Clinical Chemistry and Center for Clinical Research, Centralsjukhuset, Karlstad, Sweden; 3 Karlstad Central Hospital, Rosenborgsgatan 9, 652 30 Karlstad, Sweden

**Keywords:** Blood specimen collection, Urine specimen collection, Traumatic brain injuries, S100 calcium-binding protein beta subunit

## Abstract

**Purpose:**

Traumatic brain injury causes morbidity and mortality worldwide. S100B is the most documented emergency brain biomarker and its urine-assay might be advantageous because of easier sampling. The primary aim was to evaluate urine S100B’s ability to rule out intracranial hemorrhage. Secondary aims included S100B temporal pattern for 48 h post-trauma and chemical properties of urine that affect urine S100B.

**Methods:**

Patients with head trauma were sampled for serum and urine S100B. Patients who were admitted for intracranial hemorrhage were sampled for 48 h to assess S100B-level, renal function, urine-pH, etc.

**Results:**

The negative predictive value of serum S100B was 97.0% [95% confidence interval (CI) 89.5–99.2%] and that of urine S100B was 89.1% (95% CI 85.5–91.9%). The specificity of serum S100B was 34.4% (95% CI 27.7–41.6%) and that of urine was 67.1% (95% CI 59.4–74.1%). Urine-pH correlated strongly with urine S100B during the first 6-h post-trauma. Trend-analysis of receiver operator characteristics of S100B in serum, urine the arithmetic difference between serum and urine S100B showed the largest area under the curve for arithmetic difference, which had a negative predictive value of 93.1% (95% CI 89.1–95.8%) and a specificity of 71.8% (95% CI 64.4–78.4%).

**Conclusion:**

This study cannot support ruling out intracranial hemorrhage with urine S100B. Urine-pH might affect urine S100B and merits further studies. Serum and urine S100B have poor concordance and interchangeability. The arithmetic difference had a slightly better area under the curve and can be worth exploring in certain subgroups.

## Introduction

Traumatic brain injury (TBI) is a significant burden to healthcare systems worldwide. In the United States, it resulted in 2.5 million emergency department visits, 282,000 hospitalizations, and 56,000 deaths in 2013 [1]. Between 70 and 90% of TBIs can be classified as mild and pose a diagnostic dilemma, since the majority of the patients have no dangerous injuries, while the minority will have intracranial hemorrhage, and some of these patients will need neurosurgical intervention [2, 3]. The seriously injured patients may initially show no symptoms to differentiate them from the patients with benign injuries.

To identify patients with intracranial hemorrhage, an in-hospital observation or a computerized tomography (CT) of the head can be employed. Both strategies show similar outcomes [4]. Liberal head-CT scanning has emerged as a more cost-effective option than observation. However, there may be a risk of radiation-induced neoplasms, especially in the younger population [4]. A biomarker that could rule out intracranial hemorrhage and reduce the need for CT would probably be beneficial in reducing both radiation and costs. Several biomarkers for TBI have been suggested, with serum S100B as the most studied one [5]. When analyzed within 6 h (h) after trauma, serum S100B has a negative predictive value (NPV) of more than 99% [6]. The Scandinavian Neurotrauma Committee Guidelines have incorporated serum S100B into its algorithm and stipulate that certain patients whose serum S100B is below the cutoff of 0.1 μg/l can be safely discharged without CT [7].

We envisage that the potential benefits of using urine protein S100B level in the Emergency Department would be to distinguish between the patients with minor TBI (Glasgow Coma Scale 14–15) that need radiological workup and the ones that can be safely discharged—much like serum protein S100B is used today in some countries. It is possible there might be some benefits if urine S100B could be used past the 6-h limit of serum protein S100B but when more than 6 h after trauma have passed, the risk of large brain injury due to intracranial hemorrhage is probably significantly smaller. Further benefits include eliminating the need for phlebotomy and minimizing the staff time needed for sampling.

Questions about missing epidural hematomas in patients with normal S100B have arisen. Because of the small number of these patients in the studies, the risk has not been properly established [8]. There are reports of patients presenting with an epidural hematoma and a serum protein S100B level below the cutoff [9, 10].

Protein S100B (21 kDa) originates mainly from astrocytes but is also found in other cells, such as chondrocytes, Schwann cells, adipocytes, and malignant melanoma cells. It regulates calcium homeostasis and has neurotrophic effects in nanomolar concentrations and neurotoxic effects in micromolar concentrations [11]. It is secreted from astrocytes into the cerebrospinal fluid, passes through the blood–brain barrier and the glymphatic system into the blood, and is eliminated by renal filtration. It has a half-time of 25–97 min (min) [12].

S100B is a stable biomarker and is not readily susceptible to analytical error because of preanalytical handling and hemolysis [13]. Thus far, no study has examined if it can be accurately measured in urine as well and no studies have evaluated the precision of the urine S100B assay. Furthermore, no studies have investigated the temporal profile of urine S100B in adults with intracranial hemorrhage, and elevated levels can possibly be detected in urine longer than in serum. This might allow the analysis of S100B in urine for a longer time after trauma than the current 6-h limit stipulated for the analysis of serum S100B. A biphasic pattern of S100B levels in serum after trauma has been observed; therefore, it is reasonable to investigate the relationship between serum and urine samples [14].

The primary aim was to evaluate the ability of urine S100B to rule out intracranial hemorrhage in patients with head trauma. The secondary aims were to establish temporal profile of urine S100B over 48 h after trauma of patients with intracranial hemorrhage due to head trauma and to study how renal function and the chemical properties of urine affect urine S100B.

## Materials and methods

The study was conducted in Helsingborg General Hospital, Helsingborg, Sweden. The catchment area included 350,000 people. It was a non-randomized, prospective cohort study involving adult patients presenting to the emergency department with head trauma.

Population 1 was selected for the study on S100B serum and urine levels of patients with isolated head trauma. This group included patients who were 18 years or above and seeking emergency medical care due to isolated head trauma.

Population 2 was selected for the study on the serum and the urine S100B temporal profiles of patients with intracranial hemorrhage. This group included patients who were 18 years or above and had CT-verified intracranial hemorrhage due to head trauma. The patients who underwent neurosurgical intervention were excluded, because they were transferred to another hospital. All patients were supposed to be sampled upfront at registration, regardless of the time that had elapsed from their trauma to 1, 2, 3, 4, 5, 6, 8, 12, 16, 24, and 48 h after registration. Plasma creatinine, plasma cystatin C, estimated glomerular filtration rate (eGFR), urine-pH, urine osmolality, and urine creatinine were also analyzed at each sampling point. The eGFR was calculated using the mean values of creatinine and cystatin C.

In both populations, patients under 18 years were excluded, as well as those with multitrauma, as it might lead to false positive S100B levels due to extracerebral S100B (mainly from adipocytes and chondrocytes) [15]. Other data, such as time of injury, sex, current medication, and so on, were obtained retrospectively through a review of the patients’ medical records.

The venous blood samples (4 ml) of both populations 1 and 2 were taken through a venipuncture of the upper extremity or an indwelling catheter into regular serum-separating tube vacutainers that contained a serum-separating gel without additives. The samples were initially stored at room temperature for up to 2 h and transported to the laboratory, as in daily practice. They were then centrifuged at 2200*G* for 30 min. The serum samples of population 1 were immediately analyzed by different operators, as in daily practice. The serum samples of population 2 were frozen at − 70 °C and analyzed later in the same session by one operator.

The urine samples (5–10 ml) of population 1 were collected in empty sample tubes through voluntary voiding, a preexisting indwelling catheter, or a catheter inserted in the emergency department, per the order of the physician in charge. The urine samples of population 2 were collected in empty sample tubes through an indwelling open catheter (which kept the bladder empty) and sampled straight from the catheter tube to the test tube, not allowing for any significant bladder time. They were refrigerated at 4 °C until they were transported to the laboratory and centrifuged for 30 min at 2200*G*. The samples from population 1 were analyzed immediately. The samples from population 2 were frozen at − 70 °C and analyzed in the same way as the serum samples. Precision analysis was performed 10 times on one serum sample and one urine sample of the first six patients in population 2.

The S100B assay was done on a Cobas e411 S100 electrochemiluminescence assay (Roche Diagnostics, Stockholm, Sweden) in the in-hospital laboratory (accredited by the Swedish Board for Accreditation and Conformity assessment). The results were uploaded into the hospital’s electronic medical record database. The assay had a processing time of 18 min and a detection limit of 0.005 μg/l in serum. The manufacturer did not provide any detection limit for urinary analysis. The within-assay coefficient-of-variation of serum (CV) was 1.8%, and the imprecision was 3.1% for concentrations ranging from 0.08 to 2.13 μg/l [16]. Plasma cystatin C, plasma creatinine, urine osmolality, and urine creatinine were analyzed on Cobas e411 according to the standard protocol.

## Ethics

Authors declare no conflicts of interest. Study was approved by the Regional Ethical Review Board in Lund, Sweden. Specific national laws were observed.

## Statistics

The data was analyzed in SPSS version 25 for Mac. Histograms were used to test for normal distribution. Central tendencies were presented as means (1.96 × standard deviation) when the variables were parametric and as medians [interquartile range (IQR)] when they were non-parametric. Spearman’s correlation coefficient (*ρ*) was used to assess the correlation of the non-parametric variables. Linear regression was used to create explanatory equations for the dispersion of scatter plots. The receiver operator characteristics (ROCs) were analyzed with fixed cutoffs; the trends of all available samples without specific cutoffs, were also analyzed. The areas under the curves (AUCs) were stated with a 95% confidence interval (CI). Both serum and urine S100B samples were considered independent variables and compared with each other with the Mann–Whitney *U* test. The statistical significance level was set at *p* < 0.05. The CV (%) was calculated as (the standard deviation of the samples)/(the mean of the samples).

## Results

### Precision analysis of serum and urine S100B

The mean S100B concentrations were 0.151 (± 0.225) μg/l in serum and 0.067 (± 0.200) μg/l in urine. The mean CV% in all six serum samples was 1.30 (± 1.078). The mean CV% in urine samples was 3.16 (± 3.114), as listed in Table 1.

**Table 1:** Precision analysis of serum and urine S100B

Sample #	Analysis	Mean (10 samples)	SD	CV % = (SD/mean) × 100
1	S-S100B	0.292	0.004	1.35
	U-S100B	0.046	0.001	2.08
2	S-S100B	0.034	0.001	2.18
	U-S100B	0.030	0.001	2.47
3	S-S100B	0.095	0.001	0.66
	U-S100B	0.019	0.001	3.86
4	S-S100B	0.301	0.003	1.08
	U-S100B	0.274	0.004	1.34
5	S-S100B	0.083	0.001	1.65
	U-S100B	0.014	0.001	5.83
6	S-S100B	0.103	0.001	0.89
	U-S100B	0.022	0.001	3.37

Assay performed on six individual patient samples, with ten within-day analyses of each sample

### Serum and urine levels of S100B

#### Population 1

Over a 6-month period in 2018, in total, 512 patients with head trauma were included in population 1. Of the 512 patients, 73 had medical records, where the time of the trauma was impossible to ascertain. No venous samples were rejected by the laboratory, but eight urine samples were rejected, because the wrong sampling tube was used. Of the remaining 431 patients, three had missing venous S100B samples. Matching urine and blood samples were obtained in 243 of these patients. Their mean age was 60.8 years (± 44.96 years). Of the 243 patients, 13 (5.4%) had intracranial hemorrhage. The CT frequency was 151/243 (62.1%). Unconsciousness was confirmed in 58/243 (23.9%) cases, but in 38/243 (15.6%) cases, it could not be ascertained. Amnesia was present in 71/243 (29.2%) cases. All patients were awake when they arrived in the emergency room (13–15 on the Glasgow Coma Scale). Of the 243 patients, 37 (15.2%) took warfarin or an oral anticoagulant, 24 (9.9%) took 75 mg of aspirin, 2 (0.8%) were administered clopidogrel, 3 (1.2%) were given a combination of aspirin (75 mg once daily) and ticagrelor (90 mg twice daily), and 1 (0.4%) had a serious bleeding disorder.

**Fig. 1:**
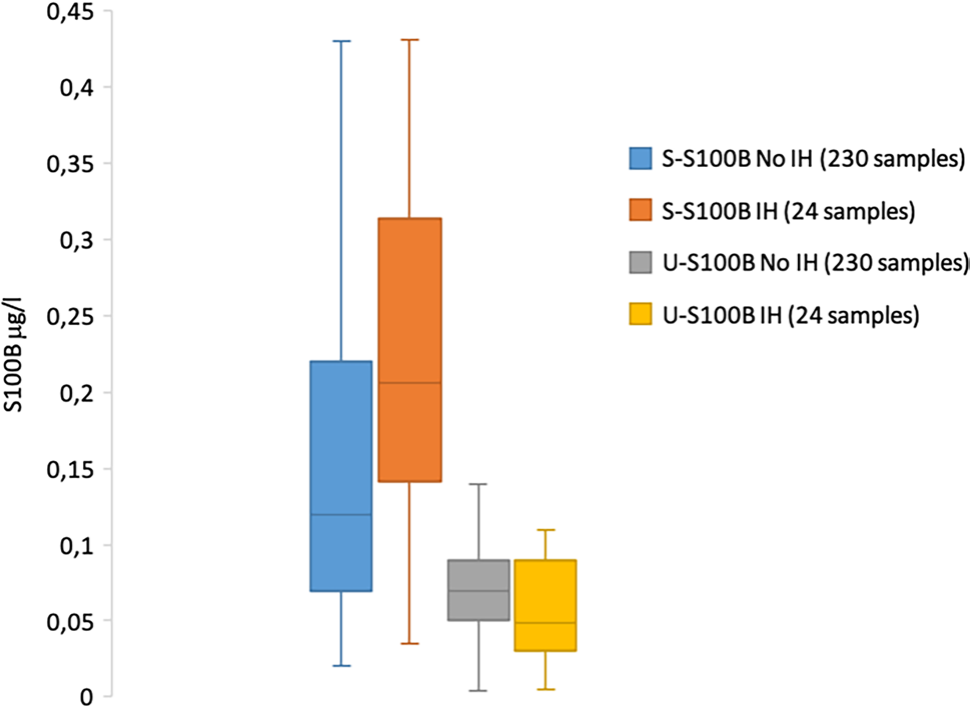
Box-and-whiskers plot of all S100B samples from patients with and without intracranial hemorrhage and patients with intracranial hemorrhage, sampled within 6 h from trauma. All samples from patients with and without intracranial hemorrhage are from population 1, and all samples taken from patients within 6 h from their trauma are from population 2. *IH* intracranial hemorrhage, *S* serum, *U* urine

The median S-S100B of the 230 patients without intracranial hemorrhage was 0.12 (0.07–0.22 IQR) μg/l, and their median U-S100B was 0.07 (0.05–0.09 IQR) μg/l (41.7% lower than the serum level). The median S-S100B of the 13 patients with intracranial hemorrhage was 0.18 (0.12–0.35 IQR) μg/l, and their median U-S100B was 0.08 (0.045–0.10 IQR) μg/l (66% lower than the serum level). The median S-S100B of all 243 patients was 0.13 (0.07–0.23 IQR) μg/l, and their median U-S100B was 0.07 (0.05–0.09 IQR) μg/l (46.2% lower than the serum level). See Fig. 1 for the box-and-whisker plot of the data, together with the data from population 2.

#### Populations 1 and 2, patients with intracranial hemorrhage

During a 3-month period in 2017, in total, 13 patients with CT-verified intracranial hemorrhage were included in population 2.

In total, 11 samples from population 2, drawn from 6 patients within 6 h from their trauma, were available for analysis. The median serum S100B was 0.22 (0.18–0.29) μg/l; the median urine S100B was 0.039 (0.0230–0.0435) μg/l. The difference in the median serum S100B in these 11 samples from population 2, compared with the 13 patients with intracranial hemorrhage in population 1, was 0.22–0.18 μg/l = 0.04 μg/l (*p* = 0.794). The corresponding difference in the median urine S100B was 0.039–0.08 μg/l = − 0.041 μg/l (*p* = 0.010). The patients whose samples were taken over time in the intensive care unit (population 2) had levels of serum S100B that did not statistically differ from those in population 1 and had significantly lower levels of urine S100B. See Fig. 1 for the box-and-whiskers plot of the data from population 2, sampled within 6 h of their trauma, together with the data from population 1.

**Fig. 2:**
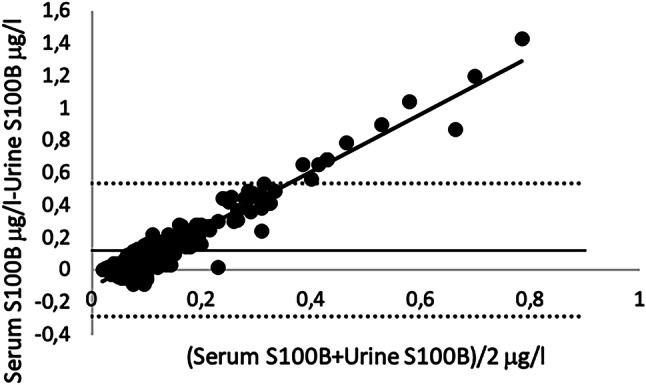
Bland–Altman plot of serum and urine S100B. In the figure, 239 matched samples are included, and four samples are excluded. Regression line: intercept = −  0.114 (95% CI −  0.155–0.073), slope = 1.747 (95% CI 1.628–1.865), *R*^2^ = 0.778

The Bland–Altman plot of S100B in the serum and the urine samples from population 1 (shown in Fig. 2) displays a mean bias of 0.12 (± 0.411) μg/l. The limits of agreement range from − 0.287 to 0.536 μg/l.

**Fig. 3:**
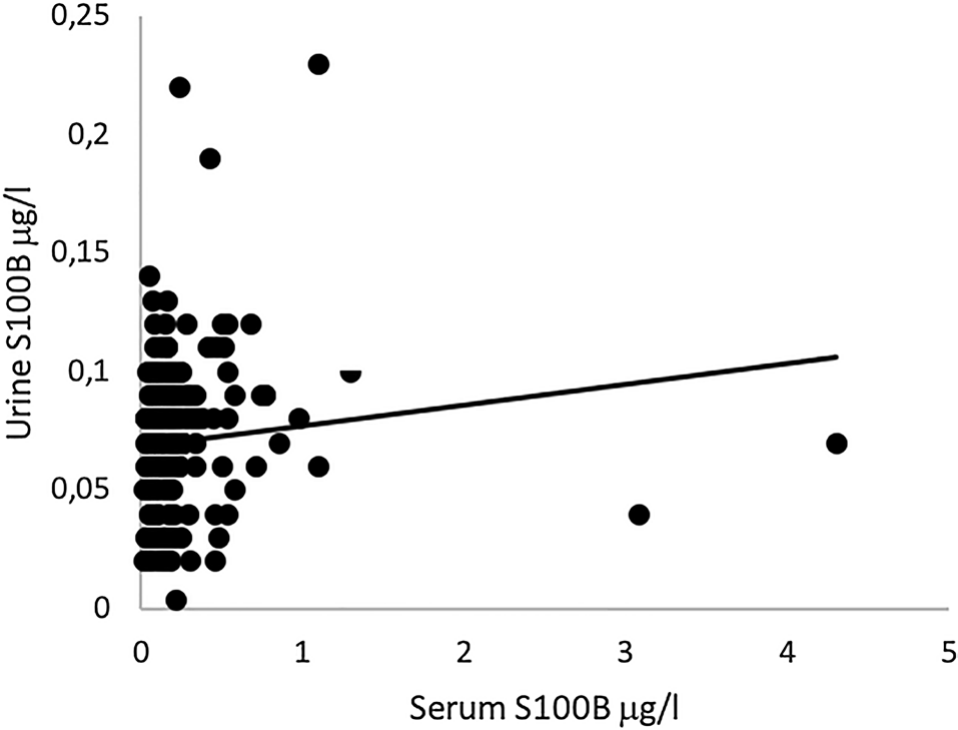
Correlation plot of serum and urine S100B samples within 6 h or less from trauma to serum sampling. In the figure, 199 matched samples are included, and 2 samples are excluded. Intercept = 0.069 (95% CI 0.0633–0.0738), slope = 0.009 (95% CI −  0.003 to 0.020), *R*^2^ = 0.012, Spearman’s *ρ* = 0.229 (*p* = 0.001)

Figure 3 shows the correlation plot of serum S100B and urine S100B in the samples from population 1, with Spearman’s *ρ* = 0.207 (*p* = 0.001).

One patient with intracranial hemorrhage had a serum S100B value of less than the clinical cutoff of 0.10 μg/l, measured within the 6-h limit.

### Temporal profile of serum and urine levels of S100B in patients with intracranial hemorrhage (population 2)

**Fig. 4:**
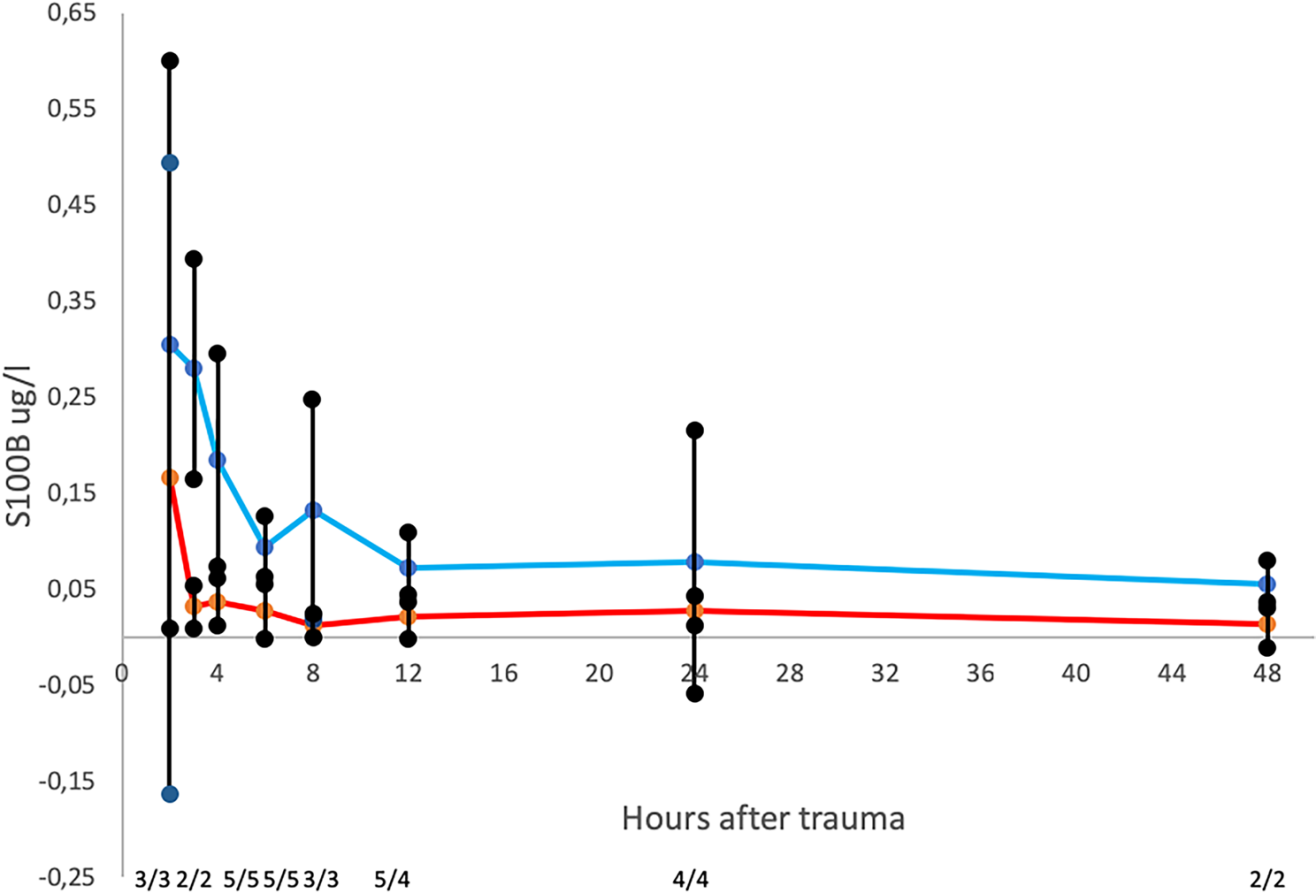
Mean serum and urine S100B temporal profile after CT-verified traumatic intracranial hemorrhage. The figure shows 42 matched serum and urine samples; 6 patients are included in the analysis. Median age = 68 years (IQR 46–69); blue line—serum S100B, red line—urine S100B. The numbers at the bottom of the figure (e.g., 3/3) are the numbers of the serum/urine samples included to calculate mean value at each point in time

In total, 13 patients were included in population 2 to establish the temporal profile and analyze the correlation and the agreement of S100B between serum and urine. Of these 13 patients, 6 had 2 or more valid samples at 0, 4, 6, 8, 12, 24, and 48 h that were included in the statistical analysis; in total, 43 matched samples were included. The urine S100B level dropped faster than the serum level and had less variation than the serum level. There was no significant elevation of the urine S100B compared with the serum after the first 6 h (Fig. 4).

### Properties of kidney function and urine composition affecting S100B levels in urine

Plasma creatinine, plasma cystatin C, eGFR, urine osmolality, and urine creatinine did not correlate with the urine S100B levels.

**Fig. 5:**
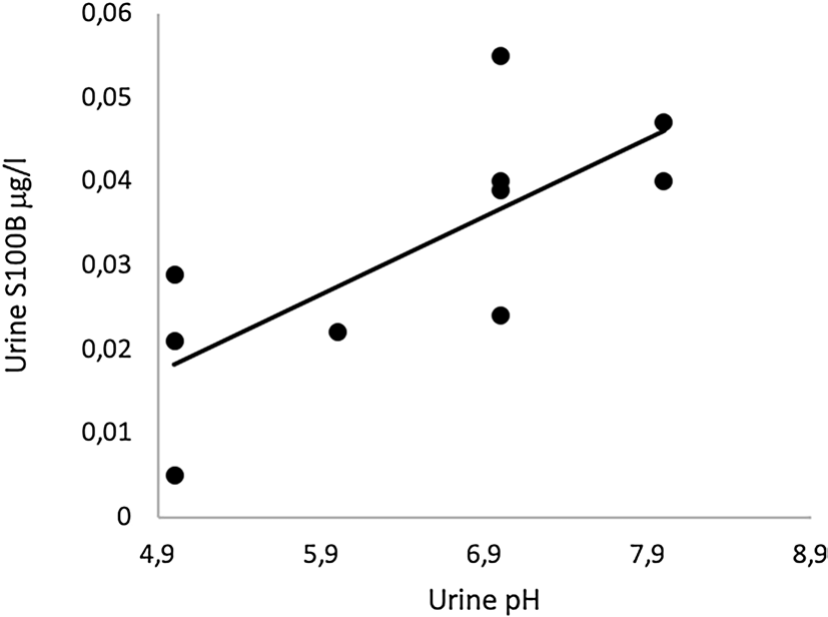
Correlation plot of urine-pH and urine S100B within 6 h after trauma. One outlier is excluded. Ten samples are included in the graph. Regression line: intercept = −  0.03 (95% CI −  0.07–0.02), slope = 0.009 (95% CI 0.002–0.016), *R*^2^ = 0.551, Spearman’s *ρ* = 0.778 (*p* = 0.008)

The only analysis of blood and/or urine that showed a correlation to urine S100B was urine-pH, measured within 6 h after trauma. Spearman’s *ρ* = 0778 (*p* = 0.008). See Fig. 5. After 6 h from the trauma, the correlation could no longer be observed.

### Receiver operator characteristics of relationship between serum and urine samples and intracranial hemorrhage

In population 1, the serum S100B in the samples drawn 6 h or less from trauma (12/201 patients with intracranial hemorrhage) had the best cutoff at 0.1 μg/l (AUC = 0.589, 95% CI 0.436–0.741, *p* = 0.304). In population 1, the best cutoff for urine S100B, sampled within 6 h from trauma (10/180 patients with intracranial hemorrhage), was 0.09 μg/l (AUC = 0.635, 95% CI 0.454–0.816, *p* = 0.151).

In the combined populations 1 and 2, for the serum S100B samples drawn 6 h or less from trauma (23 samples from patients with intracranial hemorrhage), the AUC was 0.628 (95% CI 0.523–0.734, *p* = 0.044). For the urine S100B samples drawn 6 h or less from trauma (21 samples from patients with intracranial hemorrhage), the AUC was 0.502 (95% CI 0.371–0.633, *p* = 0.977).

**Fig. 6:**
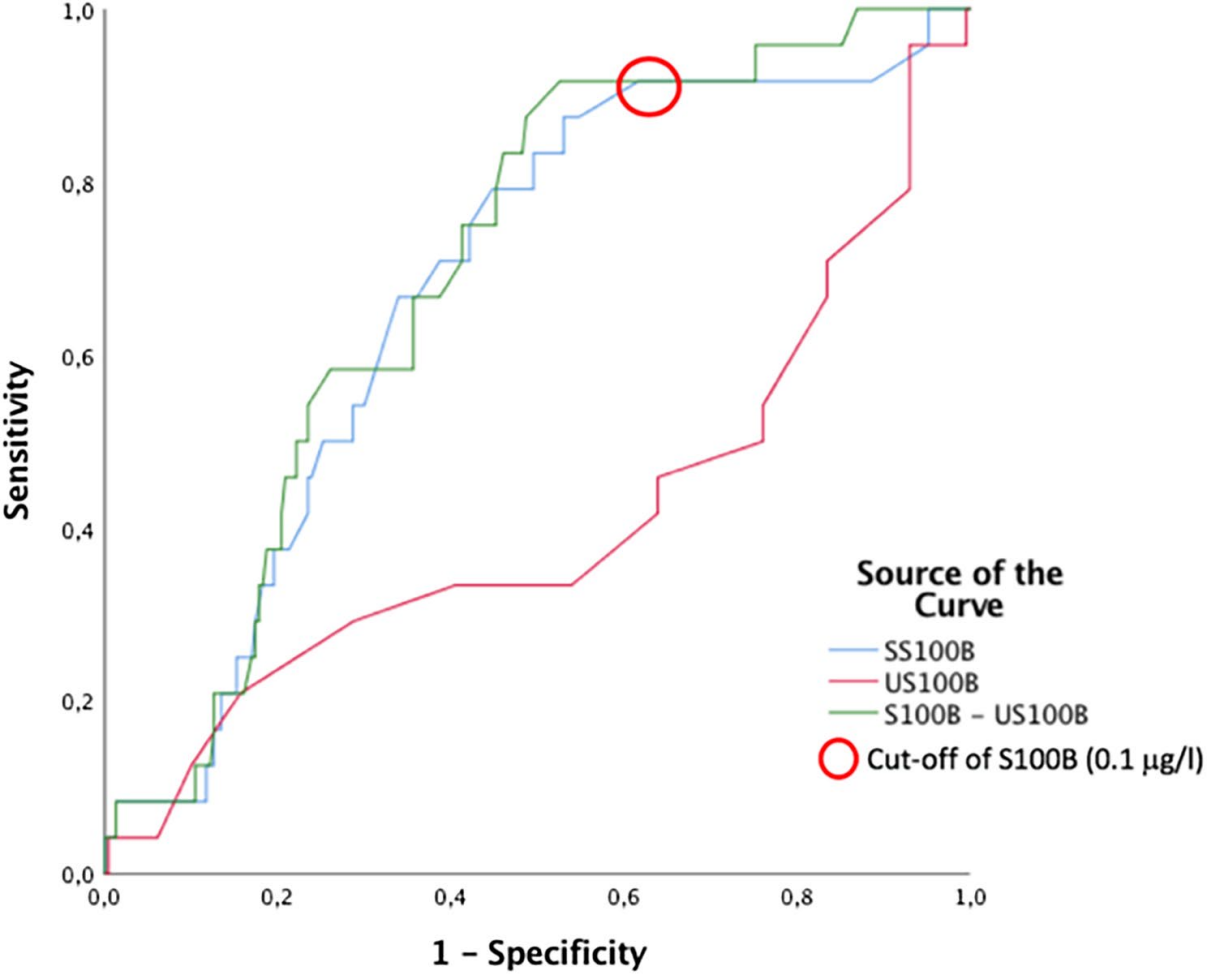
Receiver operator characteristics’ curve of population 1 and population 2 samples within 6 h after trauma. In total, 191 samples are included in the analysis, with 21 samples from patients with intracranial hemorrhage. Area under curve (AUC) of SS100B = 0.657 (95% CI 0.542–0.772, *p* = 0.019), AUC of US100B = 0.386 (95% CI 0.232–0.540, *p* = 0.089), AUC of SS100B-US100B = 0.686 (95% CI 0.581–0.790, *p* = 0.006). Diagonal segments are produced by ties

The arithmetic difference, the ratio, and the log-ratio between the paired serum S100B and urine S100B samples were analyzed. Only the arithmetic difference between these samples had a larger AUC than the two samples analyzed individually when tasked to rule out intracranial hemorrhage. The ROC trend analysis of serum S100B, urine S100B, and the arithmetic difference between serum S100B and urine S100B in populations 1 and 2 within 6 h from trauma showed the largest AUC for the arithmetic difference. See Fig. 6.

In population 1, both serum and urine levels of S100B were higher when sampled ≤ 6 h from trauma compared with > 6 h from trauma. This was the case in both cohorts with and without intracranial hemorrhage, except for one patient with intracranial hemorrhage (see Table 2).

**Table 2:** Time between trauma and sampling of serum S100B and urine S100B

Intracranial hemorrhage	Serum S100B level, µg/l							
	< 6 h (h)				> 6 h			
	*N*	Median	25th percentile	75th percentile	*N*	Median	25th percentile	75th percentile
No	189	0.13	0.08	0.24	41	0.09	0.05	0.13
Yes	12	0.18	0.12	0.35	1	0.29	–	–

Intracranial hemorrhage	Urine S100B level, µg/l							
	< 6 h				> 6 h			
	*N*	Median	25th percentile	75th percentile	*N*	Median	25th percentile	75th percentile
No	170	0.07	0.05	0.09	60	0.05	0.03	0.08
Yes	10	0.09	0.05	0.10	3	0.05	–	–

The cutoff levels of the arithmetic difference between 0.08 and 0.20 μg/l were tested at 3–6 h after trauma. In some instances, the arithmetic difference had a higher NPV than the serum sample but was never statistically significant. Neither were there any significant differences in the NPV between serum S100B with a cutoff of 0.1 μg/l and urine S100B with a cutoff of 0.09 μg/l, as well as their difference with a cutoff of 0.15 μg/l up to 6 h after trauma. See Table 3.

**Table 3:** Comparison of serum and urine S100B assay performance up to 6 h after trauma in populations 1 and 2

Assay	Sensitivity	Specificity
S-S100B ≥ 0.10^a^	91.3% (95% CI 71.96–98.93%)	34.4% (95% CI 27.7–41.6%)
U-S100B ≥ 0.09^b^	33.3% (95% CI 14.6–57.0%)	67.1% (95% CI 59.4–74.1%)
S-S100B—U-S100B ≥ 0.15^b^	57.1% (95% CI 34.0–78.2%)	71.8% (95% CI 64.4–78.4%)
	Negative predictive value	Positive predictive value
S-S100B ≥ 0.10^a^	97.0% (95% CI 89.5–99.2%)	14.5% (95% CI 12.6–16.6%)
U-S100B ≥ 0.09^b^	89.1% (95% CI 85.5–91.9%)	11.1% (95% CI 6.2–19.2%)
S-S100B—U-S100B ≥ 0.15^b^	93.1% (95% CI 89.1–95.8%)	20.0% (95% CI 13.9–28.0%)

^a^212 samples

^b^191 samples

## Discussion

The CV for the S100B assay in the serum samples matches the CV stated by the manufacturer. The absolute variation in the urine samples is the same as that in the serum samples (Table 1). However, the CV is higher in the urine samples, which is probably because the assay is designed to achieve the best precision at clinically important serum values, in this case, the clinical cutoff of 0.1 μg/l. If the cutoff for ruling out intracranial hemorrhage in urine would be established at a very low level of urine S100B, this higher CV might be a problem. The median urine S100B (0.07 μg/l) of the patients’ samples taken in the emergency department is very close to the manufacturer-specified interval of 0.08–2.08 μg/l, whose precision is good. Thus, the S100B assay in urine is feasible from an analytical perspective. Protein S100B, being very insensitive to preanalytical handling and hemolysis, further supports this conclusion [13].

The median levels of S100B taken within 6 h from population 2 with intracranial hemorrhage are higher, and the median levels of the urine samples are lower in population 2 than in population 1. The urine values are expected to be higher as well, reflecting the higher serum levels. The low number of samples makes it difficult to draw any conclusions. However, because of its statistical significance, this finding might merit future studies. The analysis of the temporal profile of the serum and the urine S100B levels of patients with intracranial hemorrhage shows that both levels drop fast within the first 6 h. The urine levels remain very low and have less variation than the serum levels during the observed 48 h.

The Bland–Altman plot with a mean bias of 0.12 μg/l also supports conclusion that urine S100B and serum S100B are not interchangeable. The limits of agreement are far too wide for an analysis that is supposed to be used at a fixed rule-out value (Fig. 2). However, the regression model can explain 77.8% of the variance in the samples, which can indicate a rather small or at least a constant bias from a preanalytical error and from variation in the assay method. The plot has a large positive slope, indicating a larger mean difference at higher S100B levels in the samples. The present study’s precision analysis with the highest tested serum concentration of 0.301 μg/l can neither support nor refute this finding, because high values of S100B are not included. There might be analytical bias, but high S100B levels in urine could likely be analyzed with good precision as well. Instead, it is possible that high levels of serum S100B are not as well reflected in urine compared with lower levels. This assumption is supported by Hallén et al., who showed that elevated serum S100B levels in children with TBI were not equally elevated in urine [17]. However, they used a different assay and had previously shown that different assays were not interchangeable [18].

The correlation plot shows a large dispersion between the samples and poor linearity (Fig. 4). This result is supported by the very low correlation coefficient. The positive slope of regression indicates less correlation in higher concentrations of serum S100B.

Urine S100B has been studied as a prognostic marker for asphyxic newborns [19]. Several studies have explored the correlation between urine and blood S100B but have focused on the outcome in severe TBI [20]. Schültke et al. studied urine S100B as a discriminator for intracranial hemorrhage, but because of the assay’s lower detection limit (0.02 μg/l), they could not draw any definite conclusions [32]. Hallén et al. explored the correlation between blood and urine S100B in the pediatric population and found that it was low [17].

The difference in the urine S100B levels between patients with and without intracranial hemorrhage is small, and the difference in their serum S100B levels is larger. This difference cannot be explained in the current study but is most likely not because of analytical issues. It is theoretically possible that bladder time and/or the chemical composition of urine, such as its osmolality, pH, and so on, may influence the S100B level. The strong correlation between urine-pH and urine S100B samples within 6 h from trauma may indicate that urine S100B levels are affected by the urine-pH. This correlation is not observed throughout 48 h because of the very low levels and the small variations in urine S100B after 6 h. Only a few studies outline the pathophysiology of low urine-pH and how it affects urine proteins [21]. Reagan et al. showed that the urine-pH did not significantly affect the dipstick urine protein assay in rats, but no human studies have been published [22]. This correlation needs to be further studied in an in-vitro setting, specifically examining how different levels of urine-pH affect S100B concentrations.

It is conceivable that many of the false positive serum levels might be produced by a swift outflux of S100B caused by the primary injury that occurs at the moment of impact. The urine S100B of a patient with head trauma but who does not have an intracranial hemorrhage might reflect this first S100B surge. The outflux of S100B from the central nervous system might be sustained if there is secondary injury to the brain. The secondary injury can be produced by ischemia, inflammation, and local edema as consequences of an intracranial hemorrhage. The rationale for using the arithmetic difference between serum and urine is that by subtracting the urine S100B level from the serum S100B level, it might be possible to reduce the implication of the primary peak of S100B that only represents the injury caused by the impact, not the elevation of S100B caused by the actual intracranial hemorrhage. This would theoretically generate less false positives and increase specificity but not necessarily affect the test’s NPV and the ability to safely rule out intracranial hemorrhage. If the decrease in false negatives is large enough, it would generate a larger AUC, which might be the explanation for the larger AUC in the present study.

The arithmetic difference had a larger AUC than serum S100B. However, neither urine S100B nor the arithmetic difference can be recommended as a biomarker for intracranial hemorrhage in the clinical setting, because the sensitivity is too low. It could possibly be beneficial if urine S100B and the arithmetic difference would be applied to a subgroup of patients, similar to the current S100B guidelines presented by Undén et al. (patients with mild head injury and low risk of intracranial hemorrhage) [7]. The AUCs reported in the present study are similar to the previously reported AUC for serum S100B (0.690) [23]. Because no patients with epidural hematoma were included in the study, nothing can be said about urine S100B in these patients.

False negative serum S100B samples that are obtained within 6 h after trauma are rare but do occur, and a false negative result is the major drawback of biomarkers. The specificity of serum S100B has previously been reported at 34%, which is similar to the finding in the current study and is exceeded by both urine S100B and arithmetic difference [24]. However, specificity is not the most important feature of a biomarker that is used to rule out intracranial hemorrhage.

## Conclusions

The present study does not support using urine S100B to rule out intracranial hemorrhage. However, the pH of urine seems to impact urine S100B and should, therefore, be studied further. Serum and urine S100B are not interchangeable due to their poor correlation and poor agreement. The arithmetic difference between serum and urine S100B had a slightly better AUC than both serum and urine samples. The arithmetic difference between serum and urine S100B can be worth exploring in a larger prospective study, where the sampling will be directed toward certain subgroups of patients.
